# A Systematic Review of Evidence for the Clubhouse Model of Psychosocial Rehabilitation

**DOI:** 10.1007/s10488-016-0760-3

**Published:** 2016-08-31

**Authors:** Colleen McKay, Katie L. Nugent, Matthew Johnsen, William W. Eaton, Charles W. Lidz

**Affiliations:** 1 Department of Psychiatry, Program for Clubhouse Research, Systems and Psychosocial Advances Research Center, University of Massachusetts Medical School, 55 Lake Avenue North, Worcester, MA 01655 USA; 2 Maryland Psychiatric Research Center, University of Maryland School of Medicine, PO Box 21247, Baltimore, MD 21228 USA; 3 Department of Sociology, Worcester State University, 486 Chandler Street, Worcester, MA 01602 USA; 4 Department of Mental Health, Johns Hopkins Bloomberg School of Public Health, Room 880B, 624 North Broadway, Baltimore, MD 21205 USA; 5 Department of Psychiatry, Systems and Psychosocial Advances Research Center, University of Massachusetts Medical School, Worcester, MA 01655 USA

**Keywords:** Evidence, Evidence based practice, Psychosocial rehabilitation, Clubhouse

## Abstract

The Clubhouse Model has been in existence for over sixty-five years; however, a review that synthesizes the literature on the model is needed. The current study makes use of the existing research to conduct a systematic review of articles providing a comprehensive understanding of what is known about the Clubhouse Model, to identify the best evidence available, as well as areas that would benefit from further study. Findings are summarized and evidence is classified by outcome domains. Fifty-two articles met the selection criteria of Randomized Clinical Trials (RCT’s), quasi-experimental studies, or observational studies for domains of employment (N = 29); quality of life/satisfaction (N = 10); reductions in psychiatric hospitalization(s) (N = 10); social relationships (N = 10); education (N = 3); and health promotion activities (N = 2). RCT results support the efficacy of the Clubhouse Model in promoting employment, reducing hospitalization(s), and improving quality of life. Quasi-experimental and observational studies offer support in education and social domains. The findings from this review indicate that Clubhouses are a promising practice but additional studies using rigorous methods that report the strength of the outcomes are needed to evaluate Clubhouse programs with fidelity to the Clubhouse Model.

**Electronic supplementary material:**

The online version of this article (doi:10.1007/s10488-016-0760-3) contains supplementary material, which is available to authorized users.

## Introduction

The *Clubhouse Model* of Psychosocial Rehabilitation has been in existence for over 65 years, has worldwide presence, and has positively affected thousands of individuals diagnosed with serious mental illness (SMI) (Propst 1997). Prior to the development of Fountain House, a group of patients discharged from Rockland State Hospital formed the self-help group We Are Not Alone (WANA) to offer each other support. WANA reorganized at the end of 1947 and Fountain House started operating as a social club in New York City (NYC) in 1948 (Anderson 1999). Fountain House began incorporating aspects of the Clubhouse Model as it is known today (e.g. emphasis on a workday) with the arrival of John Beard in 1955 (Doyle et al. 2013).

Fountain House remained the sole Clubhouse until the National Institute of Mental Health funded the National Clubhouse Training Program in 1977 (Propst 1997). Today, 326 Clubhouses located in 33 countries and 36 states in the U.S. are affiliated with Clubhouse International. While the Clubhouse Model has been broadly disseminated, this manuscript is the first to provide a comprehensive review of the evidence base for the Clubhouse Model.

## The Clubhouse Model

Clubhouses are intentionally formed, non-clinical, integrated therapeutic working communities composed of adults and young adults diagnosed with SMI (members) and staff who are active in all Clubhouse activities (Dougherty 1994; Doyle et al. 2013; Macias et al. 2001). Clubhouse membership is open to anyone who has a history of mental illness. Membership is voluntary and without time limits. Being a member means that an individual is a critical part of the community and has both shared ownership and shared responsibility for the success of the Clubhouse.

Clubhouses are strengths-based, emphasize teamwork, and provide opportunities for members to contribute to the day-to-day operation of the Clubhouse through the *Work*-*ordered Day*, with members and staff working side-by-side as colleagues to run the program (Doyle et al. 2013). The work-ordered day parallels the typical business hours of the working community where the Clubhouse is located. Clubhouses strive to help members participate in mainstream employment, educational opportunities, community based housing, wellness, or health promotion activities, reduce hospitalizations or involvement with the criminal justice system, and improve social relationships, satisfaction, and quality of life.

Basic principles of the Clubhouse Model include the belief that every member has individual strengths to recover from the effects of mental illness sufficiently to lead a personally satisfying life; and a belief that work, and work-mediated relationships are restorative. Fundamental elements of the Clubhouse Model include the right to membership and meaningful relationships; the need to be needed; choice in type of work activities; choice in staff selection; and a lifetime right of re-entry and access to all Clubhouse services (Beard et al. 1982; Macias et al. 1999). A key component of the model includes employment at prevailing wages in the wider community through Transitional Employment (TE), Supported Employment (SE), and Independent Employment (IE). Each type meets the federal definition of competitive employment, the positions are mainstream, and pay at least minimum wage. Transitional Employment (TE) positions are time-limited, part-time opportunities, usually 6–9 months in duration. The Clubhouse develops and maintains a relationship with the employer, provides onsite training and support, and coverage by a Clubhouse staff or member in the case of an absence. TE positions “belong” to the Clubhouse and members will have as many opportunities to participate in TE as needed. The employer leaves the decision as to who will fill the TE(s) to the Clubhouse.

Clubhouse Supported Employment (SE) is not designed to be time-limited and jobs may be full or part-time. The Clubhouse provides support both on and off-site upon the member’s request. While the Clubhouse often has some relationship with the employer in SE, these jobs are not “owned” by the Clubhouse and there is a “competitive element” to the interview.

Clubhouse Independent Employment (IE) is distinguished from Clubhouse SE by the lack of a formal relationship between the employer and the Clubhouse and the absence of on-site supports. Members participating in IE have participated in a fully competitive interview.

Most Clubhouses offer some type of supported education (SEd), and a Clubhouse standard requires that Clubhouses assist members “to further their vocational and educational goals by helping them take advantage of adult education opportunities in the community”. Mowbray et al. (2003) estimated that the majority of supported education programs available for adults with SMI were provided by Clubhouses. Educational supports typically include counseling or mentoring, tutoring group supports, and/or group related classroom preparation and mobile supports (Mowbray et al. 2005).

Additional components of the model include evening, weekend, and holiday activities; and decision-making and governance. Clubhouses also provide a variety of other supports through the “functions of the house” which include helping with entitlements, housing and advocacy, promoting healthy lifestyles, as well as assistance in finding quality medical, psychological, pharmacological and substance abuse services in the community reach-out (contacting/visiting members that have not been attending the Clubhouse) (Clubhouse International 2015).

## Clubhouse Affiliation, Accreditation, and Standards

Clubhouses that wish to join the international Clubhouse network pay dues and affiliate with Clubhouse International (formerly ICCD). Clubhouse International oversees a set of rigorous quality standards (International Clubhouse Standards) (Propst 1992) that serve as operational guidelines and form the basis of the Clubhouse Accreditation process. All Clubhouses affiliated with Clubhouse International strive to meet the International Clubhouse Standards and have sent a team of members and staff to participate in intensive training on the Clubhouse Model.

The International Clubhouse Standards were developed in 1989 to address the need for quality assurance and dissemination of information, and to define what constitutes a Clubhouse (Propst 1992). The standards define and strengthen Clubhouse values and practice, (Propst 1997; Jarl 1992; Norwood 1992). The Clubhouse Standards are organized by eight categories: Membership, Relationships, Space, the Work-Ordered Day, Employment, Education, Functions of the House, and Funding, Governance, and Administration.

Clubhouse Accreditation, established in 1992, is a symbol of quality and a demonstration of commitment to the International Clubhouse Standards (Macias et al. 1999). Approximately half of the Clubhouses currently affiliated with Clubhouse International have received Clubhouse Accreditation.

There are two measures of clubhouse fidelity described in the literature: the Clubhouse Fidelity Index (CFI) (Lucca 2000) and the Clubhouse Research and Evaluation Screening Survey (CRESS) (Macias et al. 2001). The Clubhouse Research and Evaluation Screening Survey (CRESS) is a brief instrument designed to measure operational fidelity and predict clubhouse readiness for accreditation and performance in model outcomes (Macias et al. 2001). The CRESS confirms whether a program has an acceptable level of compliance with the International Clubhouse Standards. The CFI is a brief instrument that assesses program implementation of clubhouse components and differentiates programs at three levels of clubhouse fidelity.

The focus in this review is on Clubhouses affiliated with Clubhouse International because many different types of services use the word “Clubhouse” although they may not attempt to adhere to the International Clubhouse Standards or have fidelity to the Clubhouse Model.

## Systemic Data Collection

Clubhouses track outcomes through the Clubhouse Profile Questionnaire (CPQ) (Clubhouse International 2016). The CPQ is an electronic database that gathers program-level information concerning practices, characteristics, concerns, and performance outcomes of Clubhouse programs. The CPQ replaces earlier versions of the ICCD Clubhouse Survey. Areas addressed in the CPQ include: Funding, governance and administration; membership; staffing and staff credentials; work unit structure; employment; housing; services provided; participation in Clubhouse training; and research activities. CPQ summary data were used in several documents (Gorman 2012, 2015; Kelliher 2006; McKay et al. 2007) including SAMHSA’s Mental Health United States 2010 and Behavioral Health 2012 publications [Substance Abuse and Mental Health Services Administration (SAMHSA), 2012, 2013]. A variety of Clubhouse characteristics obtained from the CPQ are presented in Table 1.

**Table 1:** Characteristics of clubhouses participating in an annual survey of clubhouse programs

	United States Clubhouses		Non-United States Clubhouses	
	Averages			
	Accredited (N = 61)	Non-accredited (N = 41)	Accredited (N = 18)	Non-accredited (N = 39)
Average daily attendance	47.4	31.6	46.1	27.0
Active membership	162.0	99.3	158.4	94.4
Attempts to meet Clubhouse standards	100.0 %	78.0 %	100.0 %	90.0 %
Annual budget	$709,841	$454,159	$894,245	$681,014
Belongs to a Clubhouse coalition	82.0 %	56.0 %	83.0 %	49.0 %
Interior space (sq. ft.)	7836	5816	3302	2524
Number staff (FTE’s)	8.7	6.0	10.3	5.0
Clubhouse has consumer staff	25.0 %	33.2 %	13.8 %	12.7 %
Length of operation (years)	20.9	15.4	18.1	14.7
Club offers transitional employment	98 %	83 %	73 %	82 %
Club offers supported employment	97 %	83 %	87 %	76 %
Club offers independent employment	98 %	97 %	93 %	79 %
Member (active) to staff ratio	19.5:1	18.5:1	17.8:1	17.9:1
Cost per member per day	$41.48	$43.44	$84.40	$90.62
Cost per member per year	$4776	$5065	$11,183	$8217

Data obtained from an annual survey of Clubhouses: http://iccd.org/Clubhouse_survey.html

Despite the longevity of the model, several literature reviews included few studies describing the effectiveness of the Clubhouse Model or of outcomes related to Clubhouse participation (Bond et al. 1997; Crowther et al. 2001, 2010; Drake et al. 2003). These reviews were constrained to studies that examined employment outcomes only, did not examine all types of Clubhouse employment, and/or randomly assigned individuals to program models. None of these reviews examined the scope of existing evidence for the Clubhouse Model across a range of outcomes from research designs including RCT’s, quasi-experimental studies, and observational studies. Here we generate a more comprehensive summary of evidence for the Clubhouse Model by examining the full extent of results for six outcome domains in a variety of research designs with varying levels of evidence.

## Methods

This article reviews the extent of the evidence for the Clubhouse Model. The goals of this study are to make use of the existing research to provide a comprehensive understanding of what is known about the Clubhouse Model, identify and synthesize the best evidence available, and identify areas that need further study. The authors target research in six outcome domains including: (1) employment including TE, SE, and IE, (2) hospitalization/recidivism, (3) quality of life/satisfaction, (4) social relationships, (5) education, and (6) health promotion activities. These six domains were selected because they have published literature, they represent the most commonly reported quantitative outcomes from Clubhouses, and they reflect the core goals of the Clubhouse Model. Each of these domains is described below. In some cases, literature that describes the impact of the Clubhouse Model on other domains is lacking. For example, we were unable to find published data on the impact of housing supports provided by Clubhouses.

Searches were made for the years 1948 through 2015, using PubMed, Google Scholar, OVID, EBSCO and Dissertations Express and the keywords “Clubhouse”, “Fountain House”, “Transitional Employment”, and “Mental Health”, or “Mental Illness” to identify articles for each domain. The authors also reviewed the holdings of a Clubhouse reference library, which is an inventory of published literature on the Clubhouse Model. The resource library is available on Clubhouse International’s web site (Clubhouse International 2015) and includes a list of citations for published manuscripts, dissertations, and grey literature on the Clubhouse Model. Our review included research publications, doctoral dissertations, and government reports. This approach follows the practice of several evidence-grading systems including the Cochrane Collaboration (The Cochrane Collaboration 2016; Higgins and Green 2011), the Guide to Community Preventative Services (Community Preventive Services Task Force 2016), and the Campbell Systematic Reviews (The Campbell Collaboration 2016).

## Inclusion and Exclusion Criteria

Our inclusion criteria included documentation or verification that the program described in the literature was operating as a Clubhouse (adhering to the Clubhouse Standards, achieved Clubhouse Accreditation, etc.), that the study used rigorous research designs, and described findings within at least one of the six domains that we identified. We provide additional details about the inclusion criteria below.

The initial search yielded over two hundred and fifty articles. However, many of these publications were descriptions of the model or Clubhouse practices, personal narratives describing member experiences or testimonials from Clubhouse experts, or reported on process outcomes such as the formation of a new group. While these articles and narratives are useful and informative, as they provide some of the foundations for research on Clubhouses, we have excluded them from the manuscript due to restricting the evidence to rigorous research designs described below (see Appendix A).

Some articles described program level outcomes such as Clubhouse costs, did not separate Clubhouse data from the rest of the sample, or did not offer quantitative data on the outcomes of interest described above. Additionally, some Clubhouses did not report following the International Clubhouse Standards; did not have fidelity to the Clubhouse Model; or were missing key components such as Transitional Employment or the Work-Ordered Day. These articles are cited in the reference list but not described further. Only studies that reported quantitative results in one or more of the six outlined domains were investigated further. In order to be included in the review, studies of Clubhouses in Tables 2 and 3 had to have been accredited by Clubhouse International and/or reported following the International Clubhouse Standards at the time of the study, or had reported completing comprehensive training on the Clubhouse Model. Since the International Clubhouse Standards were not instituted until 1989, studies conducted prior to the creation of the Clubhouse standards were excluded unless the studies were conducted at Fountain House, or at Clubhouses where the Clubhouse under study reported following the Fountain House Model, or where key personnel had participated in comprehensive Clubhouse training. In some cases, the authors were unable to determine if some Clubhouses were accredited or affiliated with Clubhouse International so the authors excluded these articles from the review. In cases where the Clubhouse’s fidelity and adherence to the International Clubhouse Standards and/or accreditation status was not specified the authors reviewed historical Clubhouse directories (annual registries of dues paying member clubhouses that strive to follow the Clubhouse Standards) from Clubhouse International in order to eliminate or verify articles for inclusion in their review.

**Table 2:** Evidence from experimental or quasi-experimental studies on the Clubhouse model

Citation		Outcome	Comparison group	Sample	Significance
Randomized Controlled Trials					
Beard et al. (1963)**	Proportion Re-hospitalized: Clubhouse participants had a lower proportion re-hospitalized at every time interval during the 2 year follow-up study. By 9 months, 46 % of controls and 28 % of Clubhouse members had been re-hospitalized.		Persons referred to other community services	352 (274 Clubhouse, 78 comparison)	p < 0.01
Beard et al. (1978)**	Proportion Re-hospitalized: A smaller proportion of Clubhouse members were re-hospitalized at 6, 12, and 24 months. The subgroup of Clubhouse members receiving 2 years of reaching out services had a lower proportion re-hospitalized at 5 years.		Persons referred to other community services	333 (252 Clubhouse, 81 comparison); all had been hospitalized in the prior 4 months	p < 0.01 (6 months) p < 0.05 (12 months) p < 0.02 (24 months) p < 0.02 (5 years, subgroup)
	Days Hospitalized: Over 9 years of follow-up, Clubhouse members spent less time in the hospital (39 vs. 50 months).				p < 0.05
	Proportion Re-hospitalized: A smaller proportion of Clubhouse members were re-hospitalized at 6, 12, 18, and 24 months.		Persons referred to other community services	74 (40 Clubhouse, 34 comparison); all had been hospitalized in the prior 4 months	p < 0.05 (6, 12, 24 months) p < 0.02 (18 months)
Gold et al. (2016)	Global Quality of Life: Clubhouse participants reported greater global quality of life improvement, particularly with the social and financial aspects of their lives, as well as greater self-esteem. Clubhouse participants who worked a competitive job reported greater service satisfaction compared to other Clubhouse participants.		PACT	167 (83 Clubhouse, 84 PACT)	P < .05 Effect Size = 0.00 Self Esteem: (est. = 1.02, SE = 0.41, t = 2.50, p = 0.01) (M = 24.5, SD = 4.7, n = 38 vs. M = 21.3, SD = 5.9, n = 36);
Johnsen et al. (2004)*^a^	Employment Days: Clubhouse members in transitional employment positions had a greater number of days worked compared to persons in jobs set aside for mentally ill persons.		Persons receiving Assertive Community Treatment (PACT)	175	p < 0.01, N = 17
	Employment—hourly wage: Clubhouse members in transitional employment positions had greater wage than persons employed in set-aside jobs.				p < 0.01, N = 17
Macias et al. (2001)*^a^	Employment rate: A similar percentage of both groups became employed (66 % in PACT and 70 % in Clubhouse).		PACT	166 (80 Clubhouse, 86 PACT)	p = 0.581
	Employment tenure: There were no significant differences in work duration between Clubhouse and PACT.				Data not reported
Macias et al. (2006)*^a^	Employment rate: There was no difference in employment rates between PACT (64 %) and Clubhouse (47 %) or in days to first job.		PACT	174 (58 Clubhouse, 63 ACT)	p = 0.06 (employment rate) p = 0.492 (days to first job)
	Employment hours: Clubhouse members worked more total hours (median 494 vs. 234).				p = 0.040
	Employment wage: Clubhouse members earned more (median $3456 vs. $1252).				p = 0.023
	Employment duration: Clubhouse members worked longer (median 199 days vs. 98 days).				p = 0.048
Schonebaum et al. (2006)*^a^	Employment Placement: No significant differences between groups (60 % Clubhouse vs. 74 % PACT.		PACT	170 (86 Clubhouse, 84 PACT)	p = 0.052
	Employment Duration: Clubhouse members worked more weeks per job than PACT (mean of 21.8) vs. 13.1 weeks).				CI = 9.8-16.4; *x* ^2^ = 6.37, df = 1, p < 0.01
	Employment Wage: Clubhouse members earned more (mean of $7.38/h vs. $6.30/h).				Clubhouse CI = $6.74-$8.02, Pact CI = $6.03-$6.58;, *x* ^2^ = 7.72, df = 1, p < 0.01
	Employment Positions Worked: No significant differences between groups (2.2 Clubhouse vs. 2.1 PACT).				Estimated at p = 0.676 (full data not provided)
Schonebaum and Boyd (2012)*^a^	Employment Duration: Greater Clubhouse Work-Ordered Day participation prior to employment was associated with greater employment duration. Work-Ordered Day hours prior to and during competitive employment had a small significant correlation with each other.		PACT	43	t (36) = 3.38, p < .01 r(41) = .30, p < .05
Matched Designs					
Henry et al. (1999)*		Hospitalizations: High Clubhouse attendees experienced a nominally greater decline in number of hospitalizations comparing the first to third years of enrollment.	Persons matched on gender and case management enrollment date)	862 (509 Clubhouse, 353 comparison)	p = 0.080
		Emergency Mental Health Encounters: In the first year, high attending Clubhouse members had more emergency encounters than low attending members and the matched group. During the second and third years, high attendees had a significantly greater decline in emergency encounters compared to low attendees and the matched group.			p = 0.012 (year 1, high vs. low attendees) p < 0.001 (year 1, high attendees vs. matched) p < 0.001 (all comparisons, years 2 & 3, high attendees vs. low attendees and high attendees vs. matched)
Mowbray et al. (2009)**		Quality of Life: Clubhouse members reported a higher quality of life controlling for demographics, symptomatology, and disability.	Geographically matched centers	31 Clubhouses and 31 Consumer Drop In Centers (>1800 consumers)	Coefficient^b^ = .15 p = 0.048
		Recovery Orientation: A greater proportion of Clubhouse members self-reported to be in recovery from mental illness (71 % vs. 52 %).			Coefficient^b^ = .43 p = 0.004 OR = 1.54
Tsang et al. (2010)**		Employment Rate: A greater proportion of Clubhouse members were employed during the 6-month follow-up (24 % vs. 2 %).	Age and sex-matched individuals from a regional outpatient clinic	92 (46 pairs)	p < 0.01
		Quality of Life: At baseline, Clubhouse members had lower physical health-related QOL. At three and six month follow-ups, Clubhouse members showed improvements in physical, psychological, social relationships and environmental QOL domains.			p < 0.01 (baseline) Cohen’s d effect size = <0.01, p < 0.001 for all follow-ups (Author reports these results are not significant after Bonferroni correction)
Warner et al. (1999)*		Social Support: A greater proportion of Clubhouse members reported having close friends (92 % vs. 62 %) and someone to rely on when they needed help (100 % vs. 63 %).	Group of patients matched on diagnosis, age, sex, psychiatry history, and previous service use.	76 (38 pairs)	p = 0.002 (close friend) p < 0.001 (someone to rely on)
		Quality of Life: Clubhouse members reported better QOL for finances, legal/safety, and global well-being.			(legal/safety t = 2.18, df = 69, p < 0.01) (finances t = 2.18, df = 69, p < .05), global well-being t = 2.4, df = 74, p < .05)
		Hospitalization: During the first 6 months, a higher proportion of Clubhouse members were hospitalized (13 % vs. 3 %).			p = 0.108
		Employment Rate: A higher proportion of Clubhouse members were employed (45 % vs. 34 %).			p = 0.327
		Employment hours: Clubhouse members worked less hours.			p = 0.003

Evidence on Effects of Clubhouse

* Clubhouse Accredited by Clubhouse International

** Clubhouse Adheres to Standards and/or had Fidelity Check

^a^Participants from a single study, (the EIDP), were used in separate analyses for all five publications: (Macias et al. 2001) n = 166, (Macias et al. 2006) n = 174, Schonebaum et al. (2006) n = 170, (Schonebaum and Boyd 2012; Schonebaum et al. 2006) n = 43, (Johnsen et al. 2004) n = 175, (Gold et al. 2016), n = 167

**Table 3:** Classification of Clubhouse Effects on Range of Outcomes*

Domain	# Studies	# Supportive	Multiple or Single site RCT’s	Quasi-experimental Designs	Observational studies
Hospitalization	10	6^a^	3	2	5
Employment	29	15^b^	6	4	19
Quality of Life/Satisfaction	10	6^c^	1	4	5
Social Relationships/Inclusion	10	8^d^	0	8	2
Education	3	0	0	0	4
Health Promotion Activities	2	1^e^	0	0	2
Totals	64*	30	10	18	36

* Nine articles provide evidence in multiple domains

# Supportive

^a^Beard et al. (1963, 1978), Booth (1994), Crowther et al. (2010), Grinspan (2015), Henry et al. (1999)

^b^Baker (2012), Barry (1982), Beckel (1998), Donnell (2001), Gold et al. (2016), Hancock et al. (2015), Johnsen et al. (2004), Macias et al. (2001a, b), Macias et al. (2006), Schonebaum et al. (2006), Schonebaum and Boyd (2012), Stein et al. (1999), Tsang et al. (2010), Yau et al. (2005)

^c^Boyd and Bentley (2005), Gold et al. (2016); Jacobs (1999), Jung and Kim (2012), Mowbray et al. (2009), Warner et al. (1999)

^d^Adler (1976), Biegel et al. (2013a, b), Booth (1994), Carolan et al. (2011), Mowbray et al. (2005), Spence (2014), Warner et al. (1999)

^e^Pelletier et al. (2005)

Each article was placed into one of five classes of evidence based on research designs in the Cochrane Approach (2011): (A) multisite Randomized Clinical Trials (RCT’s), (B) single site RCT’s or controlled comparisons, (C) observational studies, (D) expert consensus or testimony, and (E) personal narratives (staff and/or member). Restricting the criteria for inclusion to published articles in the first three classes of evidence (A–C) from Clubhouses with fidelity or adherence to the model reduced the final sample to fifty-two individual papers or publications. In some cases, there were multiple publications from the same RCT.

The authors created summary charts of the evidence for each domain, organized by source, sample size (N), design type, service(s) provided, duration of study, study findings (including how the outcome was measured), whether the study was published, strength of evidence grade, and comments. A written summary with significant findings and implications of each study was created from information organized within each chart (Supplementary Table 1). Fourteen of the publications provided results from experimental designs (Table 2). The final sample of fifty-two articles provides outcome evidence in the six domains as follows: (1) work and/or employment (N = 29), (Baker 2013; Barry 1982; Beckel 1998; Booth 1994; Crowther et al. 2010); Donnell 2001; Dorio et al. 2002; Gold et al. 2016; Gregitis et al. 2010; Hancock et al. 2015; Henry et al. 2001, 1999; Jacobs and DeMello 1996; Johnsen et al. 2004; Kelliher 2006; Macias et al. 2001; Macias et al. 2001; Macias et al. 1995; Macias et al. 2006; Malamud and McCrory 1988; McKay et al. 2005, 2006; Reed and Merz 2000; Schonebaum and Boyd 2012; Schonebaum et al. 2006; Stein et al. 1999; Tsang et al. 2010; Yau et al. 2005; (2) quality of life and/or satisfaction (N = 10) (Accordino and Herbert 2000; Boyd and Bentley 2005; Gold et al. 2016; Jacobs 1999; Jung and Kim 2012; Mowbray et al. 2005, 2009; Rosenfield and Neese-Todd 1993; Stein et al. 1999; Warner et al. 1999); (3) hospitalization (N = 10) (Accordino and Herbert 2000; Beard et al. 1978; Beard et al. 1963; Booth 1994; Crowther et al. 2010; Grinspan 2015; Henry et al. 1999; Malamud and McCrory 1988; Mowbray et al. 2005; Wilkinson 1992); (4) social relationships/social networks (N = 10) (Adler 1976; Biegel et al. 2013a, b; Booth 1994; Carolan et al. 2011; Gumber 2011; Mowbray et al. 2005; Pernice-Duca 2008; Spence 2014; Warner et al. 1999; (5) education (N = 3) (Dougherty et al. 1992; Unger and Pardee 2002; Weiss et al. 2004); and (6) wellness/health promotion activities (N = 2) (Onkon et al. 2015; Pelletier et al. 2005). In some cases individual papers or publications provided evidence in multiple domains (N = 9).

## Results

Results for each domain are presented in order from research with evidence from RCT’s to research with evidence from observational studies. A summary of studies with evidence from Randomized Clinical Trials or Quasi-Experimental designs with matched participants within each of the six domains is provided in Table 2, while a summary of the main findings of all 52 publications can be found in Supplement 1. Findings from Table 2 are classified by type and level of evidence in Table 3, based upon significance and frequency of findings that provided support for the Clubhouse Model.

## Employment

Twenty-nine articles addressed the impact of the employment supports (TE, SE, and IE) provided by the Clubhouse Model. Ten articles describe employment outcomes from an RCT or studies with matched participants, and nineteen describe employment outcomes from observational studies.

One RCT compared a program for assertive community treatment (PACT) and a Clubhouse as part of a multi-site randomized controlled trial of supported employment, called the Employment Intervention Demonstration Program (EIDP), which ran between 1995 and 2000 (Macias et al. 2006). Admission criteria for this study were age 18 or older, a DSM-IV diagnosis of serious mental illness, absence of severe mental retardation, being currently unemployed; and no previous PACT or Clubhouse experience. The intent-to-treat study sample (N = 175) was similar to general population descriptions of people with serious mental illness. Participants were randomly assigned to PACT or Clubhouse. Both programs had fidelity to their respective models and received regular fidelity checks throughout the study. Several papers with independent analyses of EIDP data describe outcomes that provide evidence for employment outcomes obtained through an accredited program with fidelity to the Clubhouse Model (Gold et al. 2016; Johnsen et al. 2004; Macias et al. 2006; Schonebaum et al. 2006; Schonebaum and Boyd 2012).

Macias et al. (2006) found the PACT program had greater retention of active participants than the Clubhouse (79 vs. 58 %) at 24 months (Macias et al. 2006). There were no significant differences between programs in the number of participants that attained competitive work or in the number of days to the first job during the study, but Clubhouse participants were employed more calendar days than PACT participants (264 vs. 173, p < 0.05), worked significantly more hours (784 vs. 592, p < 0.05), earned more during the study ($6202 vs. $3948, p < 0.05), and earned more per hour each week (t = 2.79, df = 65, p < 0.01) (Macias et al. 2006). Although the researchers used random assignment of individuals to programs it was impossible to blind participants or to conceal random assignment. A second analysis of EIDP data from the same study researchers found that Clubhouse members worked significantly more weeks per job (21.8 vs. 13.1), (X^2^ = 6.37 df = 1, p < 0.01) and earned significantly higher hourly wages ($7.38 vs. $6.30), (X^2^ = 7.72, df = 1, p < 0.01) (Schonebaum et al. 2006).

Schonebaum and Boyd (2012) analyzed EIDP data for forty-three participants who were active in the Clubhouse and participated in competitive employment during the study to examine the impact of participation in the Clubhouse’s Work-ordered Day on vocational outcomes. They found that participation in the Work-ordered Day prior to competitive employment was significantly associated with greater employment duration per employment cycle (duration increased by 2.3 weeks for each hour increase prior to employment (t (36) = 3.38, p < 0.01). Work-ordered Day hours prior to and during competitive employment had a small correlation with each other (r(41) = 0.30, p < 0.05). Prior work history was not significantly associated with employment duration. Positive and general psychopathology symptoms were not significantly associated with employment duration however more severe negative symptoms were significantly associated with longer average job duration (r(41) = 0.35, p = 0.02). Their findings were similar to an earlier study conducted by Macias et al. (1995) that found a strong positive correlation (r = 0.69; p < 0.0001) between tenure on Transitional Employment and attendance at Fountain House.

In a recent publication, Gold et al. (2016) found Clubhouse participants in the EIDP who worked a competitive job reported greater service satisfaction compared to other Clubhouse participants (M = 24.5, SD = 4.7, n = 38 vs. M = 21.3, SD = 5.9, n = 36).

In a secondary analysis of EIDP data, Johnsen et al. (2004) examined job process variables including intervention (Clubhouse or PACT), whether the jobs were set-aside for persons with a disability, whether jobs were temporary (e.g. jobs obtained through a temporary agency, Transitional Employment [TE] positions, etc.), or permanent by design, and whether the jobs belonged to the client or member. Johnsen et al. used three work place integration questions to create a composite workplace integration score. They found that individuals who held jobs that were set-aside specifically for persons with mental illness but were not Clubhouse TE jobs had significantly lower workplace integration (p < 0.0001), lower hourly wage (p < 0.01), fewer days employed (p < 0.01), and fewer hours worked per week (p < 0.05). Employment outcomes for persons in Clubhouse TE positions were more similar to those working in positions that were not set-aside including greater workplace integration and hourly wages compared to set-aside positions offered by Clubhouse or PACT. Clubhouse TE had the greatest number of days employed while non set-aside jobs were associated with significantly more hours worked per week. Individuals in Clubhouse TE positions and non-temporary positions such as supported or independent employment had longer job tenures compared with persons employed in PACT non-temporary positions (120.41 days for Clubhouse TE and 127.50 days for Clubhouse non-temporary positions vs. 68.51 days for PACT non-temporary positions).

## Quality of Life/Satisfaction

Ten articles describe outcomes associated with quality of life. One of the studies describing outcomes associated with quality of life was an RCT. Gold et al. (2016) tested whether competitive employment improves global quality of life in an analysis of data from a RCT that compared an accredited clubhouse and a PACT program. They found that Clubhouse participants reported greater global quality of life improvement, particularly with the social and financial aspects of their lives, as well as greater self-esteem and service satisfaction compared to competitively employed PACT participants. However, there was no overall association between global quality of life and competitive work or work duration.

Four articles describe outcomes from quasi-experimental designs and five describe findings from observational studies. Jacobs (1999) examined symptoms and satisfaction pre and post participation in a Clubhouse. The program that Jacobs examined was not affiliated with Clubhouse International but did sent staff and members for Clubhouse training. Thirty individuals with SMI aged 18–60 living in a residential treatment program in the community for at least 12 months were randomly selected from a list of clients from a community-based non-profit mental health center. Fifteen individuals from the residential treatment program participated in a Clubhouse at least 3 days/week (experimental group) and 15 individuals from the residential treatment program participated in community outings 3 days/week (control group). All participants attended the residential treatment program and participated in Clubhouse or community outings for 6 months. The Clubhouse group had a significantly higher improvement in satisfaction scores than the control group (p = 0.0214) (Jacobs 1999). The authors do not provide details regarding what the traditional residential treatment offered, what occurred during the community outings, or whether there were dropouts. Despite limitations, including a small sample size and short follow-up (6 months) Jacob’s study suggests the Clubhouse positively influences satisfaction.

Mowbray et al. (2009) examined characteristics of over 1800 users of thirty-one matched pairs of consumer-run drop-in centers (CDRI’s) and Clubhouses using random effects analysis of covariance to examine data from a study funded by the National Institute of Mental Health. CDRI’s had a significantly higher percentage of males than Clubhouses and consumers with a diagnosis of schizophrenia were more likely in Clubhouses. Clubhouse members were significantly more likely to report a higher quality of life (p = 0.048) and more likely to report being in recovery (p = 0.004, OR 1.54) than CDRI participants. There were no significant differences in hopefulness on the State Hope Scale. The study was limited by a sample located in one state, and single-item measures.

Evidence from multiple studies including two RCT’s (Jacobs 1999; Gold et al. (2016), a study with matched participants (Warner et al. 1999) and a study with matched program pairs (Mowbray et al. 2009) suggests the Clubhouse may have a positive impact on satisfaction and quality of life. However these studies have some limitations including lack of details regarding services offered in comparison groups, small sample size, or limited power.

## Hospitalization

Evidence from ten publications suggests that people who participate in Clubhouses have lower rehospitalization rates. Findings include evidence from four articles describing results from a single site RCT or studies with quasi-experimental designs, and six additional articles about observational studies.

Beard and colleagues presented findings from a two-year study of hospitalization and Clubhouse outreach (Beard et al. 1963). Study eligibility criteria included: discharge from the hospital within 4 months of intake, no prior Clubhouse contact, and a hospitalization of at least 2 months in duration in addition to Clubhouse membership requirements. At intake, participants were randomly assigned to one of three subgroups within a Clubhouse (experimental condition) or a control group. The control group subjects were referred to other services available in the community after randomization. These criteria yielded 374 subjects (274 experimentals and 78 controls). There were no significant differences between the control group and the experimental group in demographics, diagnosis, hospitalization history, medication, and treatment. All participants in the experimental conditions received typical Clubhouse services except that the length of time outreach was provided differed within subgroups of the experimental group. Rehospitalization rates and time spent re-hospitalized were significantly lower (p < 0.02) in the experimental group than they were in the control group at six and 9 months of this RCT. The authors noted that there was limited attendance and use of the Clubhouse: however, increased exposure would most likely have strengthened the effect. Crowther et al. 2010 conducted a sub-analysis of Beard’s (1963) study and found a significant difference (p = 0.026) in hospitalization rates in the first year of the study among people allocated to Clubhouse approach and those in standard community care (n = 215, RR 0.95 CI 0.49–0.96).

A subsequent publication by Beard in 1978 presented findings from nine years of follow-up on this sample (Beard et al. 1978), showing that individuals in the experimental condition averaged more time in the community before rehospitalization compared to individuals in the control group (22.5 vs. 14.6 months, p < 0.05). In the second study reported in this publication in 1978, participants were eligible if they had been out of the hospital for up to two years (Beard et al. 1978). Participants were randomly assigned to one of three experimental research sub-groups within the Clubhouse: (1) Full outreach services, (2) Outreach to non-attendees within the first month following intake, and (3) No outreach. These three groups were treated as a whole for purposes of analyses, with 40 individuals in the experimental condition and 34 in the control group. Clubhouse members receiving outreach for 2 years had significantly lower rehospitalization rates than the control group who did not receive Clubhouse services. Beard et al. note that Clubhouse participation delayed but did not prevent rehospitalization and this only became evident through their long-term study (Beard et al. 1978). While these studies describe the impact of the Clubhouse Model on hospitalization, the inadequate description of treatment provided to the control groups is a limitation.

In Mowbray et al. 2009 study examining characteristics of over 1800 users of services from one of thirty-one matched pairs of consumer-run drop-in centers (CDRI’s) and Clubhouses they found that Clubhouse members had a greater lifetime number of hospitalizations. They also found that Clubhouse members were receiving higher levels of more intensive traditional mental health services such as case management and were three times more likely to be living in a supervised setting. The authors indicate that people who may need greater structure choose a Clubhouse or are more likely to be referred to a Clubhouse by a provider within the mental health system. However, Clubhouse participants were significantly more likely to report a higher quality of life and be in recovery than CDRI participants (Mowbray et al. 2009).

Grinspan (2013) conducted a retrospective cohort study and analyzed state Medicaid claims to examine Fountain House and the use of healthcare resources among individuals who used residential rehabilitation services at Fountain House between 2010 and 2013. Grinspan found that individuals in the Fountain House cohort were consistently less likely to use the emergency department, or be admitted to the hospital compared to the comparison group.

Although there are methodological limitations with some of these studies, the evidence suggests that participation in the Clubhouse Model delays rehospitalization and reduces the likelihood of rehospitalization for persons with SMI. Similar findings are documented in published studies with varying levels of evidence including a cross sectional correlational study (Accordino and Herbert 2000), studies with matched participants and observational studies (Wilkinson 1992).

## Social Relationships/Social Inclusion

Eight quasi-experimental studies and two observational studies provide some evidence on improved social relationships for Clubhouse members. Warner, Huxley, and Berg examined quality of life, service utilization and treatment costs over 2 years comparing a group of regular Clubhouse attendees with matched participants from another town within the catchment area (non-Clubhouse users) (Warner et al. 1999). Groups were matched for age, gender, diagnosis, psychiatric history, and prior service use measured by length of contact with mental health services, resulting in 38 pairs of matched cases. The scores of Clubhouse members were significantly higher in the domains of finances, legal and safety, and global well-being in the Lancashire Quality of Life Profile (Oliver et al. 1996). Warner and colleagues report the percentages of people reporting having social relationships (92 vs. 62 %) and close friendships (100 vs. 63 %) were significantly better in the Clubhouse group than in the matched group. While this study found an accredited Clubhouse has a positive impact on quality of life and social relationships it is limited by a small sample and it is subject to performance bias given that members carried out the study and it is possible that participants will have provided socially desirable responses.

Mowbray and colleagues conducted a study of a matched sample of Clubhouses and CRDIs controlling for location, population served, program resources and operational characteristics (Mowbray et al. 2005). Variables of interest included: member involvement, services provided, and social and recreational activities. Mowbray and colleagues found significant differences between Clubhouses and CRDIs with Clubhouses providing a greater number of services (p < 0.001) but more people attending CRDIs for food and fun (p < 0.05). Clubhouses had a greater budget per consumer and provided more of the possible services asked about as compared to the consumer-run drop in centers. CRDI participants were more likely to attend for recreational/social reasons than Clubhouse members (Mowbray et al. 2005).

Biegel et al. (2013a, b) conducted a cross sectional study with 118 members in an accredited clubhouse and examined family social networks and recovery of Clubhouse members. They found that higher psychosocial functioning, greater family support, and higher positive relationship quality from the most supportive person were significantly associated with higher levels of recovery (p < 0.05).

Spence (2014) conducted an exploratory study and examined social relationships with forty-six members at one Clubhouse. Controlling for demographics and attendance, only outgoing positive comments from one member to another (p = 0.0238) and affiliation (p = 0.0187) were predictive of scores on the Maryland Assessment of Recovery in people with SMI.

Clubhouses may provide a useful vehicle for increasing social integration and social competence. Evidence supported by these studies suggests that Clubhouse participation may be beneficial in promoting social relationships although additional studies using methods that are more rigorous are needed.

## Education

Three articles (Dougherty et al. 1992; Unger and Pardee 2002; Weiss et al. 2004) with observational designs suggest Clubhouses are a potentially promising location from which to mount supported education efforts.

Utilizing data for students participating in SEd over a two-year period Dougherty et al. (1992); provide evidence of movement from SEd to employment at a single Clubhouse. A supported education program was offered that included assessments to determine reading, math, and writing skills, development of educational and career choices, coordination of services (on and off campus), and administration. SEd participants (N = 27) enrolled in community college courses (75 %), four-year colleges (14 %), and technical schools (11 %). Seventy-four percent remained enrolled after the first semester and 36.8 % remained enrolled after 18 months.

Unger and Pardee (2002) examined outcomes of 124 individuals participating in Supported Education Programs in a Mental Health Center (MHC), a Clubhouse, and a Transition to College Program for five semesters. The Clubhouse had a significantly higher percentage of students with schizophrenia (x^2^ = 6.323, p = 0.04). The mean number of credits attempted was 7.10 and the mean number of credits completed (6.43) was similar across all three programs. There were no significant differences in survival rates among sites although there was greater variation among Laurel House students. Laurel House students were also the least satisfied with school.

Weiss et al. (2004) examined a two semester curriculum developed by a Clubhouse and a community college designed to provide skills necessary for college. Sixty-nine students were served over 4 years. Weiss and colleagues indicate that students expressed satisfaction with the curriculum but they do not indicate whether all students were satisfied. After the first year of the program data was available for thirteen students. Seven students took college classes, four worked during the program, and ten worked following the program. Weiss and colleagues indicate that as many students became employed as enrolled in college.

While our search for supported education outcomes was limited to three observational studies the data suggests that offering educational supports in Clubhouses can be beneficial.

## Health Promotion Activities

All Clubhouses (N = 193) responding to a survey of Clubhouses affiliated with Clubhouse International reported offering some type of health promotion activity (e.g. health education nutrition, education opportunities for exercise, weight loss, and other activities) (McKay and Pelletier 2007). However, only two observational studies related to health promotion outcomes were available at the time of this review. Pelletier et al. (2005) found significant improvements in aerobic capacity (Wilcoxon Rank Sum Statistic 338, p = 0.0014) and emotional health (Wilcoxon Rank Sum Statistic 399.00, p = 0.046) for seventeen members from one Clubhouse that completed a 16 week structured exercise program.

Onkon et al. (2015) conducted a mixed methods study that examined the impact of a healthy lifestyles program for twenty-five members in an accredited clubhouse. Pre/post data was only available for eight members. Seven members increased their overall minutes of daily exercise, and seven experienced less anxiety with daily life stressors. However, this study was primarily descriptive and the authors did not provide significance tests.

Addressing physical health, wellness is a relatively new area of development within Clubhouses and there are only a few articles describe implementing tobacco cessation or health promotion activities within Clubhouses, without published evaluation of these interventions.

## Discussion

This systematic literature review and quantitative synthesis examined the evidence base for the Clubhouse Model of psychiatric rehabilitation looking at levels of evidence across multiple outcome domains. Recent studies of Clubhouse employment demonstrate Clubhouse members obtained employment as fast as individuals receiving employment services through other models (e.g. Program of Assertive Community Treatment [PACT]) and that members transition between the employment supports offered by Clubhouses (TE, SE, and IE) (Macias et al. 1995, 2006; McKay et al. 2006). When members move between employment types they are significantly more likely to transition from employment types that offer more supports to employment types that offer less supports (McKay et al. 2006).

Within the EIDP RCT, the earnings, job quality, and job tenure of Clubhouse members appeared superior compared to PACT participants but attrition from the Clubhouse where participation is voluntary was higher. However, findings were not as positive in a separate review. In a review of vocational rehabilitation supports, Crowther et al. 2010 examined findings from Beard et al. (1963) and found there was no difference in competitive employment for people allocated to Clubhouse approach and those in a control group (n = 215, RR 0.95 CI 0.77–1.17). A sub-analysis showed that there was insufficient evidence to determine whether the Clubhouse approach was more effective to other approaches to pre-vocational training. However, it is unclear how the results from this would translate to Clubhouses today. The Beard study was conducted shortly after Clubhouse Transitional Employment was first implemented in 1958 (Doyle, et al. 2013) and before the widespread inclusion of Supported and Independent Employment in Clubhouse supports. Today, placements in Clubhouse Transitional Employment constitute less than forty percent of all Clubhouse employment placements (McKay et al. 2005, 2006).

Despite having more research than any other domain, additional studies that examine the impact of the full range of Clubhouse employment outcomes in Transitional, Supported, and Independent Employment are needed. These studies should include comparisons with widely disseminated models of Supported Employment such as the Individualized Placements and Supports (IPS) Model. Our review identified several manuscripts published from a randomized trial of Clubhouse and PACT, yet we were unable to identify any manuscripts from a randomized trial comparing IPS to an accredited Clubhouse or a Clubhouse that adhered to the International Clubhouse Standards. Findings from the Hartford Study of Supported Employment (Mueser et al. 2004) would be relevant if the PSR program in the Hartford study had been accredited or operated with fidelity to the Clubhouse Model. Given that IPS is the most widely researched form of Supported Employment, studies comparing IPS outcomes with Clubhouse outcomes would be informative. These studies should consider Clubhouse as a separate model rather than combining it with other services as usual in order to fully understand the impact the Clubhouse has on employment outcomes.

In the domain of hospitalization multiple clinical trials suggest that Clubhouse participation reduced or delayed rehospitalization and lowered costs. Generally, there was a decline in recidivism rates and/or lengths of hospitalizations declined for Clubhouse attendees but many of these studies were conducted during the early stages at Fountain House. One recent study by Grinspan (2013) provides some data on the impact of Clubhouse participation on reducing hospitalizations but additional studies that examine the impact of Clubhouse participation on hospitalizations and reductions on health care costs are needed.

Several matched comparison studies highlight benefits of Clubhouses for members’ social relationships. Compared to participants of other mental health programs, Clubhouse members were more likely to have people in their networks that they could draw on for support. Clubhouses may provide a useful vehicle for increasing social integration and social competence, and promoting recovery. Evidence from an RCT and studies with varying levels of evidence suggests the Clubhouse also has a positive impact on satisfaction and quality of life (Jacobs 1999).

The Clubhouse offers educational opportunities and linkages with local educational institutions for members to complete or start certificate and degree programs at academic institutions and adult education programs. We found three observational studies with some data that suggests that offering educational supports in Clubhouses can be beneficial. Jones and Selim (2013) conducted a qualitative study to examine whether stigma is a barrier to education. They found that all participants (N = 6) experienced barriers to education, while some experienced stigma. Informants reported that the supported education program can help by providing encouragement and being there for them. All informants raised the importance of social interaction and support at the Clubhouse. Programs like Clubhouses that offer supports for education should consider addressing stigma as it may be a barrier for some potential students.

Findings from two observational studies suggest health promotion activities that are offered by Clubhouse programs have a positive short-term impact (Okon and Webb 2014; Pelletier et al. 2005). Additional studies of health promotion activities in Clubhouses with outcome data and longer follow-up periods are necessary.

## Limitations

The evidence from this systematic review suggests that the Clubhouse Model is effective in important domains in spite of significant methodological limitations in some studies. Many of the observational studies only report findings comparing Clubhouse members with one another and did not report findings from pre- post comparisons, making it difficult to determine whether clubhouse membership itself produced benefits. Many of the studies that we reviewed did not report effect size estimates or lacked information needed to calculate them. There were many different types of outcome measures and analysis methods reported in the studies in this review, making difficult to calculate effect sizes that would be useful in summary form or for a meta-analysis.

Clubhouses have expanded the array of supports they offer over time. Approximately one third of the studies were conducted more than 10 years ago and it is unclear whether they apply in a contemporary and specialized service environment. Programs with complex arrangements like the Clubhouse Model may not easily lend themselves to RCT’s. Researchers often examine the impact of a service provided within a particular model rather than the impact of the model as a whole. These program models do not always rely on effects of specific services and have generalized protocols and processes whose effects are difficult to isolate (Wolff 2000).

We restricted our final sample to Clubhouses that were accredited and/or followed the International Clubhouse Standards. Some studies in our initial search were conducted prior to the establishment of Clubhouse International, while others conducted later did not indicate whether the Clubhouse being examined was affiliated with Clubhouse International. Most of the Clubhouses studied in this review did not receive fidelity checks; or the researchers conducted the studies prior to the establishment of the Clubhouse Accreditation process and the development of the Clubhouse fidelity instruments. In the future, it will be important to conduct studies of accredited Clubhouse programs with strict fidelity to the model in order to evaluate the model and generalize to Clubhouses nationally or internationally. Researchers that conduct studies of the Clubhouse Model should utilize rigorous designs and measures and publish whether the “Clubhouse” studied had fidelity to the model.

## Conclusions

Administrators of mental health programs are, to an increasing extent, required to show funders that the programs they plan to implement are effective. This has been one of the driving forces behind the increasing popularity of evidence-based practices. Building the evidence base to examine the cost and impact of reductions in hospitalizations, incarcerations and other outcomes among participants in Clubhouses and other mental health settings would be beneficial to mental health administrators, particularly in an era of limited funding. Many of the existing services or programs have yet to be thoroughly investigated making it impossible to know which have the best outcomes. Unless these programs are included in research, stakeholders have no scientific way of knowing how these programs compare to existing EBPs. These models may risk elimination because of a lack of empirical research as opposed to a lack of effectiveness. Devoting resources to comprehensive research examining a wider variety of existing and innovative services like Clubhouses will increase the quantity and quality of the evidence base.

Peer driven, recovery-oriented models of psychiatric rehabilitation, such as the Clubhouse Model, are needed and expected in today’s array of supports for individuals living with mental illness. The Clubhouse Model is consistent with recovery practices with its emphasis on member choice, self-determination, community integration, equal partnerships with members and staff working side-by-side, offering hope, and helping individuals live a meaningful life. The emphasis on recovery is reflected in the growth of the literature on the Clubhouse Model in recent years (Biegel et al. 2013a, b; Conrad-Garrisi 2011; Conrad-Garrisi and Pernice-Duca 2013; Hancock et al. 2013; Pernice-Duca et al. 2013; Raeburn et al. 2014; Raeburn et al. 2016a, b; Tanaka et al. 2015).

This study provides a synthesis of the best evidence available for the Clubhouse Model. Clubhouses are a promising practice and the research supporting Clubhouses is growing, but additional studies are necessary to provide a clearer and more contemporary basis for evaluating the Clubhouse Model. Next steps include studies using rigorous methods including RCTs, studies with matched participants, or observational studies to evaluate programs with fidelity to the Clubhouse Model and develop evidence for use in a meta-analysis. Studies that examine the Clubhouse Model and other established evidence based practices would be useful. In addition, services offered by Clubhouses such as outreach or supported housing that have not been thoroughly examined would benefit from research. It will also be important to examine the impact of the Clubhouse Model where it has been adapted to serve other populations than those with severe mental illness, such as individuals diagnosed with brain injuries.

Even with these reservations, the studies in this review provide enough evidence of the Clubhouse Model’s effectiveness to assure administrators that Clubhouse programs are worthy of support as one component of a spectrum of rehabilitative services for persons with serious mental illness.

### Electronic supplementary material

Below is the link to the electronic supplementary material.

Supplementary material 1 (DOCX 51 kb)

## Appendix A: Articles Excluded

The following articles were excluded from the final review. Descriptions of the model or Clubhouse practices, personal narratives describing member experiences or testimonials from Clubhouse experts, or reported on process outcomes such as the formation of a new group (Andres 2008; Bellamy et al. 2007; Bond et al. 1999; Burt et al. 1998; Casstevens 2011a, b; Clements 2012; Coniglio et al. 2012; Conrad-Garrisi 2011; Conrad-Garrisi and Pernice-Duca 2013; Cook and Razzano 1995; Cook 1992; Delaney 1998; Dorio and Marine 2004; Dougherty 1997; Dougherty and Campana 1996; Dvir 2012; Floyd and Lorenzo-Schibley 2010; Fountain House 1999; Hiatt 1998; Gamble 2011; Henry et al. 2002; Herman et al. 2005; Hinden et al. 2009; Holter and Paul 2004; Jones and Selim 2013; Jordan and Selwyn 2008; Lee et al. 2011; Link et al. 2001; Lipe et al. 2012; Lloyd et al. 2007; Mandiberg and Edwards 2013; Marshall et al. 2011; McKay et al. 2012; McKay and Pelletier 2007; Morris 2003; Mowbray et al. 2005; Neese-Todd and Weinberg 1992; Ng et al. 2008; Pernice-Duca and Onaga 2009; Pernice-Duca 2009; Raab et al. 2014; Raeburn et al. 2015; Roth 2007; Scheid and Anderson 1995; Sheppard 2008; Snowadzky 1999; Staples and Stein 2008; Starks et al. 2000; Stoffel 2007; Tratnack and Kane 2010; Waegemakers Schiff et al. 2008; Williams et al. 2006; Wong 2010).

Articles describing program level outcomes such as Clubhouse costs and did not offer data on the outcomes of interest described above (Daniilidis 2014; Fitzgerald 2013; Fitzgerald et al. 2015a, b; Labun et al. 2012; Lesley and Livingood 2015; McKay et al. 2007; Plotnick and Salzer 2008; Pernice-Duca et al. 2015, 2010; Tanaka 2013; Tanaka et al. 2015; Tanaka and Davidson 2015; Torres Stone et al. 2015).

Clubhouses that did not report following the International Clubhouse Standards; did not have fidelity to the Clubhouse Model; or were missing key components such as Transitional Employment or the Work-Ordered Day (Cook 1992; Cook and Razzano 1995; Delaney 1998; Karp 2007; Laird and Krown 1991; Leff et al. 2004; McGurk et al. 2010; Mueser et al. 2004, 2014; Mueser and Wolfe 2010; Pirttimaa and Saloviita 2009; Schroeder 2013; Yildiz et al. 2003) were also excluded.
