# Supplementary material for: A Systematic Review of Evidence for the Clubhouse Model of Psychosocial Rehabilitation
Source: Adm Policy Ment Health. 2016 Aug 31;45(1):28–47. doi: 10.1007/s10488-016-0760-3 (PMC5756274; doi:10.1007/s10488-016-0760-3)
Supplement: Supplementary file 1 — Supplementary material 1 (DOCX 51 kb) [file 10488_2016_760_MOESM1_ESM.docx]

**Detailed Information for Publications Included in Clubhouse Review Table***

| **Citation** | **Sample Size** | **Research Design** | **Service(s) Provided** | **Study Duration** | **Outcome(s) of Interest** | **Study Findings** |
| --- | --- | --- | --- | --- | --- | --- |
| (Accordino & Herbert, 2000) | 147 (26 Clubhouse, 15 self-help, 78 social skills training, 28 vocational skills training) | Observational study comparing participants in 4 different programs | Clubhouse, Self-Help Program, Social Skills Training, Vocational Skills Training | 1 time point | Mastery, Program Satisfaction, Re-hospitalization | There were no differences by program in terms of mastery, program satisfaction or psychiatric re-hospitalization. |
| (Adler, 1976) | 50 | Observational | 4 month Transitional Employment Program | 6 months | Relationships between social participation, social competence, community involvement and realistic self-perception and employment. | Clubhouse members, after participating in a 4 month employment program, showed significant increases in social participation, social competence and community involvement. They did not experience an increase in social interaction or develop a more realistic self-perception. Social participation and community involvement were both positively associated with achieving gainful employment, while self-perception, social interaction and social competence were not associated with employment. |
| (Baker, 2012) | Quantitative survey:  168 members  23 staff  Qualitative Study:  12 members 6 staff | Observational | Usual Clubhouse services | 1 time point | Employment | Three variables in the full regression model were predictive as to whether an individual will participate in TE: Stigma/Attitudes p=.097, External Influence p=.065, and Symptoms of Mental Illness p=0.000  No significance between years of attendance and willingness to participate in TE  There is a moderate to strong relationship between the variables in the reduced regression model and Willingness to participate in TE. Total Barriers to employment was found to be significant P<.001 Total barriers was best predictor in the model for willingness to participate in TE. |
| (Barry, 1982) | 35 | Observational study | Usual Clubhouse services | 1 year | Employment, neuropsychological functioning | 56% of members who had participated for 3 months or more had at least some competitive employment, with 39% of those completing 3 months or more being able to maintain competitive employment for longer than 60 days. Aphasia and sensory perception were not associated with obtaining competitive employment. Faster time on a problem solving task, greater negative response bias and faster processing speed were predictive of competitive employment status. |
| (Beard, Pitt, Fisher, & Goertzel, 1963) | 352 (274 experimental, 78 control) | Randomized Clinical Trial, control group comprised of those referred to other community services | Social & recreational activities, work inside the Clubhouse, transitional employment, managed apartments for select participants | 3-24 months | Re-hospitalization (assessed every 3 months | For each time interval, participants in the Clubhouse program had a lower proportion hospitalized. The groups were significantly different at 6 months and 9 months post enrollment. Over the first 6 months, 35% of the control group was re-hospitalized, while only 20% of the Clubhouse group was re-hospitalized (difference significant at p<0.01). Over the first 9 months, 46% of controls were hospitalized while only 28% of the Clubhouse group was re-hospitalized (difference significant at p<0.0 |
|  |  |  |  |  | Ever Employed | 53% of controls and 60% of Clubhouse participants were ever employed after at least 1 year of the study (no significance level reported for difference) |
| (Beard, Malamud, & Rossman, 1978) | Study 1: 333 (252 Experimental, 81 control) | Randomized Clinical Trial (Clubhouse members randomly assigned to one of 3 groups that differed on their reaching-out services or a control condition) | Social & recreational activities, work inside the Clubhouse, transitional employment, managed apartments for select participants. Reaching out services (phone calls, letters, and visits) were provided to those in that experimental condition. | 9 years | Re-hospitalization | From 6 months post-enrollment to 9 years, Clubhouse participants had lower proportions re-hospitalized than controls. Differences were significant at 6 months (21% vs. 37%), 12 months (35 vs. 48%) and 24 months (44% vs. 60%). A much lower proportion of members assigned to the group that received reaching-out services for 2 years were re-hospitalized over the first 5 years. Of those participants that were re-hospitalized, participants that attended the Clubhouse spent a significantly greater time in the community prior to re-hospitalization (average of 22.5 months vs. 14.6 months for controls, p<0.05). Additionally, Clubhouse participants spent significantly less time in the hospital over the 9 years of follow up (49.6 months vs. 38.7 months, p<0.05). |
|  | Study 2: 74 | Randomized Clinical Trial (Clubhouse members randomly assigned to receive Clubhouse services or were given a referral) |  | 5 years | Re-hospitalization | Clubhouse participants had lower proportions re-hospitalized than controls. Differences were significant at 6 months (22% vs. 50%), 12 months (32% vs. 59%), 18 months (38% vs. 68%) and 24 months (42% vs. 71%). Of those that were re-hospitalized, Clubhouse participants spent an average of 15.6 months in the community before hospitalization as compared to 6.2 months for controls (p<0.01). |
| (Beckel, 1998) | 150 | Observational (Comparison of Clubhouse to Day Treatment) | All received supported employment services |  | Employment | A greater percentage of Clubhouse members were employed as compared to day treatment clients (67% vs. 39%). Clubhouse members taking clozapine were more likely to be employed. |
| (Biegel, Pernice-Duca, Chang, Chung, Min, & D’Angelo, 2013.) | 118 | Cross-Sectional Study | Usual clubhouse services | 1 time point | Social Relationships/Social Inclusion | The average personal network size was 7.92. Higher psychosocial functioning, greater family support, and higher positive relationship quality from the most supportive person were significantly associated with higher levels of recovery (p<=.05) |
| (Biegel, Pernice-Duca, Chang, & D’Angelo, 2013) | 126 | Random sample of active members from 1 Clubhouse | Usual clubhouse services | 1 time point | Social Relationships/Social Inclusion | The Clubhouse network, including staff and fellow members, comprised 9.1% of member’s social network. 62.7% of members reported having a peer network. Members with peer networks had significantly more frequent contacts with peers and were more satisfied with the relationship than non-peers (P<=.05). Peers were significantly less critical than non-peers (P<=.05).  Clubhouse visits were almost twice as high for members reporting a clubhouse network ((P<=.05). Among all respondents, being Caucasian and having better quality of social life were correlated with having a peer network while higher degree of reliance on others and more Clubhouse visits were correlated with having a Clubhouse network. |
| (Booth, 1994) | 152 | Observational (3 Clubhouses) | Usual Clubhouse Services | 1 time point (hospitalizations over the prior 6 months) | Hospitalization, Employment, Social Contact, Daily Activities | Greater program participation and positive perceptions of program relationships were associated with fewer hospitalizations. Younger members spent more days in the hospital, had higher employment status, and reported greater contacts with friends and family. Longer Clubhouse membership was associated with more social contacts. Using non-agency transportation to the program was associated with higher employment status. |
| (Boyd & Bentley, 2005) | 151 | Cross-Sectional observational study comparing drop-in centers to clubhouses | Traditional Clubhouse Services as compared to Drop-In Centers (which provide social support and assistance with social, recreational, housing, transportation & vocational problems) | 1 time point | Quality of Life | Clubhouse members reported higher general QOL. |
| (Carolan, Onaga, Pernice-Duca, & Jimenez, 2011) | 20 | Cross-sectional observational study using narrative interviews | Members had attended the Clubhouse for at least 6 months, 3/week | 1 time point | Social Support | Members identified the clubhouse as a central source of social support and a place that facilitated personal growth. |
| (Crowther, Marshall, Bond & Huxley, 2010) |  | RCT | CH (categorized as prevocational training) |  | Employment  Hospitalization | Review paper of RCTs on vocational rehabilitation. One study mentions clubhouse (Beard et al, 1963). There was insufficient evidence to judge whether CH was more effective than other approaches to prevocational training |
| (Donnell, 2001) | 245 | Cross Sectional Observational Study (17 Clubhouses) | Usual Clubhouse services | 1 time point | Employment | A greater sense of recovery and Clubhouse participation were strongly associated with being currently employed. Staff training, weekly Clubhouse attendance, availability of employment programs, length of participation, relationship with external employment programs, previous work history, sense of community and social participation were not significantly predictive of current employment. |
| (Dorio, Guitar, Solheim, Dvorkin, & Marine, 2002) | 42 | Retrospective observational study asking about previous employment experiences | Supported Employment Program | Previous 4 years of employment history | Employment (Job Retention for 1 year or more) | Members who were able to hold a job for one year or more (n=22) as compared to members who were not able to hold a job for at least one year, spent less time in the hospital, were less likely to receive financial or disability benefits, had greater ratings on functioning. More positive attitude ratings and ability to set more realistic goals were also associated with job retention. |
| (Dougherty, Hastie, Bernard, Broadhurst, & Marcus, 1992) | 46 | Observational study | Supported Education program | 18 months since program’s inception | School Enrollment and Employment | 74% remained enrolled after 1 semester (6 months), after 18 months 37% of participants remained enrolled in school. 33% gained employment independent of the Clubhouse TE program, 57% received grades of B or higher. |
| (Gold, Macias, et al 2016) | N=167  Clubhouse (N = 83)  (PACT; N = 84) | RCT | Clubhouse (N = 83) to a mobile Program of Assertive Community Treatment (PACT; N = 84) | 24 months | Employment  QOL | Random regression analyses showed that competitively employed Clubhouse participants reported greater global quality of life improvement, particularly with the social and financial aspects of their lives, as well as greater self-esteem and service satisfaction, compared to competitively employed PACT participants. However, there was no overall association between global quality of life and competitive work, or work duration. |
| (Gregitis, Glacken, Julian, & Underwood, 2010) | N= 60 | Cross-sectional observational study | Normal Clubhouse services | 1 time point | Role values (work) | There was no significant difference in the value placed on the role of work between employed and unemployed members. |
| (Grinspan, 2015) | 75 | Retrospective cohort study | Members receiving residential rehabilitation services from a Clubhouse | Data for members participating between 2010-2013 | Hospitalization | The Fountain House cohort was older (p<0.001), had a similar comorbidity burden, & was more likely to be schizophrenic (p=0.05).  Individuals in the Fountain House cohort were consistently less likely to go to emergency department or be admitted to the hospital compared to the NYC cohort. There were no clear differences between groups in the rate of outpatient care visits |
| (Gumber, 2011) | 92 members from 8 accredited clubhouses | Observational Study | Online survey looking at community integration, (internal social and psychological integration within the clubhouse community, external social community integration with non-consumers, mental health symptoms, & satisfaction with employment status. | 1 time point | Social Relationships/Social Inclusion | Member’s perceptions of self-esteem were significantly related to reports of internal integration within the clubhouse and external social integration within the larger non-consumer community. Psych symptoms were negatively correlated to measures of external social integration (r=.27, p<.01 and perceived social support r=.24, p<.05 |
| ( Hancock, Honey & Bundy 2015) | 78 | Observational Study, mixed methods | Normal Clubhouse services | 1 time point | Employment | Seven sources of meaning were attributed to most meaningful occupations: 1) positive sense of self, 2) social connections, 3) valued by others, 4) skills/personal development, 5) time use/routine, 6) financial gain, and 7) fun/pleasure.  The caring occupation was identified as most meaningful. Financial gain was associated with employment (x^2^=42.161, p<0.001) while being valued by others was cited significantly more by those for whole the career role was most meaningful (x^2^=10.292, p<0.05). |
| (Henry, Barreira, Banks, Brown, & McKay, 2001) | 138 | Retrospective review of member records | Transitional Employment | 6 years of records | Job tenure, competitive employment | Average tenure for TE was 131 days, with increasing age, longer club membership, and greater days worked per week associated with longer tenure. Disability was not associated to job tenure. 30% of members obtained competitive employment in the one year following their TE job, with greater TE hours worked predicting competitive job obtainment. |
| (Henry, Jackson, Fisher, Brown, & White, 1999) | 509 Clubhouse members, 353 controls | Retrospective review of Clubhouse and service use records of a matched control group | Normal Clubhouse services, plus all members and those in the control group were receiving case management from the state DMH | 1 year prior to Clubhouse membership plus the first 3 years after membership | Hospitalizations, Case Management and Emergency Mental Health encounters | Low attending (<1 time a month), high attending (>1 time a month) Clubhouse members and a matched control group (matched on gender and case management enrollment date) were compared on hospitalizations, case management and Emergency Mental Health encounters. High Clubhouse attenders had more case management sessions in the year prior to and the 3 years following Clubhouse enrollment compared to low attenders and controls. In the first year following enrollment, high attenders had a greater numbers of hospitalizations and emergency mental health encounters as compared to both other groups. By year 3, the high attenders had an equivalent numbers of hospitalizations as the controls and less than the low attenders. By year 3 the high attenders had less EMH encounters as compared to both other groups. |
| (Jacobs, 1999) | 30 | Randomized Clinical Trial (all lived in a residential treatment program, 15 were assigned to attend Clubhouse) | Clubhouse attendance at least 3/week vs. Community outings 3/week | 6 months | BASIS-32 and CSQ-8 assessed at baseline and 6 months | There were no significant differences in the BASIS-32 scales for either group comparing 6 month follow-up to baseline. For Clubhouse members, their consumer satisfaction of the Clubhouse significantly improved after 6 months of participation, while the control group showed no improvement in consumer satisfaction. |
| (Jacobs & DeMello, 1996) | 159 | Observational study of 2 Clubhouses, specifically members with brain injury | Transitional Employment | 15 months for Clubhouse 1, 3.5 years for Clubhouse 2 | Employment | At Moss Rehab Clubhouse, 35% of members took part in compensated work experience, with 44% remaining employed. At Dayle McIntosh Clubhouse, 24% of members participated in compensated work experience, of which 38% remain employed in competitive community employment. |
| (Johnsen, McKay, Henry, & Manning, 2004) | 175 | Randomized Clinical Trial (comparing Clubhouse to PACT) | Supported employment | 2 years | Employment characteristic | Persons who held jobs that were set-aside specifically for persons with mental illness, but were not TE jobs, had the lowest workplace integration, wage, days employed and worked hours per week. Persons in TE positions were more similar to those working in positions that were not set-aside, such that they had greater workplace integration and wage. TE had the greatest number of days worked, while non set-aside jobs were associated with the greater # of hours per week. |
| (Jung & Kim, 2012) | 521 (232 Clubhouse members from 14 Clubhouses and 289 from 15 Rehabilitation Skills Training centers) | Observational study comparing participants in Clubhouse and rehabilitation skills training programs. | Clubhouse model and the rehabilitation skills training model |  | Quality of Life | Clubhouse members reported significantly lower perceived stigma and significantly higher perceived quality of life than did the recipients of the rehabilitation skills training model. Clubhouse members specifically reported significantly higher interpersonal relationship scores than did the recipients of the rehabilitation skills training model. |
| (Kelliher, 2006) | 76 Clubhouses | Observational | Case management, medication administration | 6 month time frame | Association of case management and medication administration offered in the Clubhouse to employment outcomes | Greater case management supports were slightly associated with lower employment rates. Higher numbers of vocational supports offered by the clubhouse were positively associated with employment outcomes. No significant differences in employment outcomes were found when comparing Clubhouses that had medication services to those that did not offer medication services. Staff longevity or benefits counseling were both not associated with employment outcomes. |
| (Macias, Barriera, Alden, & Boyd, 2001) | 119 Clubhouses (71 certified, 48 noncertified) | Cross sectional survey | Usual Clubhouse services (in the certified Clubhouses) | 1 time point | Program characteristics, vocational outcomes | Certified Clubhouses had much better vocational outcomes compared to noncertified Clubhouses, such that they have a significantly larger proportion of members in TE, SE, or IE. |
| (Macias, DeCarlo, Wang, Frey, & Barreira, 2001) | 166 | Randomized Clinical Trial | Clubhouse vocational services or PACT | Up to 2.5 years | Interest in work, job placement, job tenure | Participants that identified that they were not interested in working, yet did take an employment position, had received more vocational services than those persons initially expressing interest in work. Employment rate was 70% vs. 66% (Clubhouse vs. PACT) for those persons receiving at least one hour of vocational services. |
| (Macias et al., 2006) | 174 | Multi-site Randomized Clinical Trial | Clubhouse vocational services or ACT | 18 months | Earnings, work hours | Clubhouse members had higher mean earnings and greater total work hours than persons in the ACT program. The ACT program had better service engagement and 24 month retention of participants. |
| (Macias, Kinney, & Rodican, 1995) | 295 | Observational study | Transitional Employment | 6 years | Job tenure | Considering all 720 TE positions worked over the 6 year study period, 585 were held by single members for over 3 months, and 35% were held by single members for over 6 months. Greater attendance at the Clubhouse before beginning a TEP was associated with longer duration of TE position. |
| (Malamud & McCrory, 1988) | 527 | Retrospective observational study, multiple time points | Transitional Employment | 42 months | Employment, Hospitalization | One third of participants had at least one independent employment position following a TE placement. At the end of the study 43% were independently employed or had a TE position. Length of time spent on TE was significantly related to obtaining independent employment. 28% of the sample was hospitalized at least once over 42 months, with the average time spent in the hospital being 93 days. |
| (McKay, Johnsen, & Stein, 2005) | 1702 | Observational (Annual self-report surveys) | TE, SE, IE | 3 years | Employment | Members held an average of 1.3 jobs, with 47% of employed members participating in at least one TE during the study. |
| (McKay, Johnsen, Banks, & Stein, 2006) | 2195 | Observational, multiple time points | TE, SE, IE | 4 years | Employment Transitions, level of support | Over a 4 year period, clubhouse members held an average of 1.6 jobs. Examining employment transitions, 54% remained in the same type of job in their second position as was their first job. Of those that changed employment types, movement was 1.7 times more likely to be in the direction of independent employment (from TE to SE or IE, or from SE to IE). |
| (Mowbray, Holter, Mowbray, & Bybee, 2005) | 29 clubhouses and 29 consumer drop in centers (summary stats from each center) | Cross-sectional observational study of matched centers | Consumer drop in centers focused more on social and recreational support, Clubhouses offered standard array of services | 1 time point | Characteristics of each provider (people served, agency resources), instrumental services, social and recreational services | Clubhouses had a greater budget per consumer. Clubhouses provided more of the possible services asked about as compared to the consumer drop in centers. CDI participants were more likely to attend that program for recreational/social reasons than Clubhouse members. |
| (Mowbray, Woodward, Holter, MacFarlane, & Bybee, 2009) | 31 Clubhouses and 31 geographically matched consumer run drop in centers (more than 1,800 consumers) | Cross sectional observational study of Clubhouses matched with CRDI centers | CRDI centers more focused on creating a peer support network, Clubhouses more focused on vocational aspects | 1 time point, lifetime psychiatric history was assessed | Socio-demographic characteristic of attendees, psychiatric service use, diagnosis, symptoms, quality of life | Clubhouse members were significantly more likely to be female, were less likely to have a substance abuse problem, had greater lifetime hospitalizations and were more likely to have a schizophrenia diagnosis. Clubhouse members were more than 3 times as likely to receive SSD/SSDI as CRDI center consumers. Clubhouse members were getting more intensive MH services and 3 times more likely to be living in a supervised setting. Clubhouse members report a significantly higher quality of life than CRDI center consumers, controlling for disability and symptomatology. |
| (Onkon, Webb, Zehnder, Konylski, Morrow, Reid, & Schultz-Keil, 2015) | 25 | Mixed methods | Healthy Lifestyles Program – 16 sessions – educational intervention at a Clubhouse | Intro session to gather pre-test data, then 16 weeks of 1.5 hour educational sessions then one post-test 6 week follow-up. | Wellness/Health Promotion | Pre/Post data available for 8 members. Seven members increased their overall minutes of daily exercise, seven experienced less anxiety with daily life stressors, and one increased daily intake of fruits & vegs. Primarily descriptive – no significance tests.  Members provided positive feedback about YMCA membership opportunities, and using exercise as a coping mechanism and for weight loss. Members also provided positive feedback about tobacco awareness and stress management & coping skills. |
| (Pelletier, Nguyen, Bradley, Johnsen, & McKay, 2005) | 25 | Pre and post-Test design with members serving as self-controls | Genesis Wellness Project included a structured exercise program (three 90 minute sessions a week for 16 weeks) with the overall goal of integrating health and fitness into clubhouse culture. | Pre and post intervention (16 week intervention) | Physical Health Measures, SF-36 | Among all participants that completed the program, they attended an average of 71% of scheduled exercise sessions. Participation in the 16 week program did not produce significant changes in most physical health measures, but one measure, the submaximal exercise test, did show a significant improvement. This indicates that participants gained cardiovascular capacity. Additionally, participation was associated with a significant improvement in the Mental Health subscale of the SF-36. |
| (Pernice-Duca, 2008) | 221 (from 15 Clubhouses) | Cross-sectional observational study | Usual Clubhouse services | 1 time point | Measure of Social Support Networks, Level of Social Functioning | On average, members identified having 5 people in their social network. Length of clubhouse membership and frequency of participation were not associated with the size or composition of the social network. |
| (Reed & Merz, 2000) | 36 | Observational | Developed a 2 week career planning workshop | 1^st^ 6 months | Process outcomes, % gaining employment | Over the first 6 months that the Clubhouse expanded their employment services, the employer network grew. 26 out of 36 members that participated gained employment and 25 people retained their job for 90 days. |
| (Rosenfeld & Neese-Todd, 1993) | 157 | Observational study | Clubhouse services at Club/Habilitation Services in NJ. Sought to interview all members who had participated for at least one consecutive month between 11/1988 and 11/1989 | 1 time point | Quality of Life | Empowerment from the clubhouse approach was significantly related to member’s satisfaction with their living arrangements, social relations, family relations, pre-vocational activities, safety, health, and leisure activities. Members who had more contacts in the program were more satisfied with their social relations. Members with more social contacts and spent more time in program activities were more satisfied with their leisure activities. |
| (Schonebaum, Boyd, & Dudek, 2006) | 170 | Randomized Clinical Trial | Clubhouse employment services as compared to PACT (program of Assertive Community Treatment) | 5 years | Employment (job placement, weeks worked, wage) | There were no differences between PACT and Clubhouse programs in terms of job placement rates or total jobs worked. Clubhouse members worked significantly more weeks per job than PACT clients (21.8 weeks compared to 13.1 weeks, p<0.01).Clubhouse members earned significantly higher house wages than PACT clients ($7.38 vs. $6.30 per hour, p<0.01. |
| (Schonebaum & Boyd, 2012) | 43 | Randomized Clinical Trial (this paper just reports on Clubhouse participants) | Clubhouse employment services as compared to PACT (program of Assertive Community Treatment) | 135 weeks | Employment and Work-Ordered Day participation | Participation in the Work-Ordered Day prior to being competitively employed was significantly associated with greater employment duration per employment cycle (duration increased by 2.3 weeks for each 1 hour increase prior to employment). Prior work history was not significantly associated with employment duration. Positive and general psychopathology symptoms were not significantly associated with employment duration; however more severe negative symptoms were significantly associated with longer average job duration. |
| (Spence, 2014) | 46 | Observational | Usual Clubhouse services | 1 time point | Social Relationships | Member affiliation with the clubhouse and the number of members positive comments directed at other members predict scores on the Maryland Assessment of Recovery in people with SMI (MARS) (t=2.888, p=0.00617; t=2.288, p=0.02738), regardless of clubhouse attendance which did not predict scores.  Outgoing member to member alternative emotional social support (p=0.00632), members receiving outside clubhouse alternative emotional social support (p=0.0383), and affliation (p=0.00606) were predictive of MARS scores. Controlling for demographics and attendance only outgoing positive comments from one member to another (p=0.0238) and affiliation (p=0.0187) were predictive of MARS scores. |
| (Stein, Lawton Barry, Van Dien, Hollingsworth, & Sweeney, 1999) | 59 | Cross-sectional observational study | Usual Clubhouse services vs. ACT | 1 time point | Social Networks | Clubhouse members worked more hours per week (14.4 vs. 9.2).There was no significant differences between the programs in: ratings of the importance of social support (number of friends, getting along with friends, family relationships, getting along with others), in their satisfaction with the social support they received, or in loneliness rating. |
| (Tsang, Ng, & Yip, 2010) | 92 (46 Clubhouse members matched by sex and age to patients from an outpatient clinic) | Longitudinal, case-controlled and naturalistic design | Usual Club house services vs. outpatient clinic mental health services. | 6 months. | Employment | Eleven Clubhouse members were  employed at the 6-month follow-up: 4 had attained  Independent Employment, 3 had Supported Employment, 2  had Transitional Employment, and 2 had group Transitional  Employment. One outpatient clinic patient achieved open employment. This difference was statistically significant (p ≤ 0.01). |
| (Unger & Pardee, 2002) | 124 | Observational Study | a mental health center, a clubhouse, and a community college | 5 college semesters | Education | Among all programs, 42% of supported education (SE) participants worked at some point, rising to 47% at the end of the study.  Among participants in the MHC and Clubhouse programs, there was an increase in Quality of Life associated with participation.  Among all program participants, while 85% lived independently at beginning of SE involvement, 97.8% lived independently at end of study.  Participants completed 90% of credits attempted.  Larger proportion of CH participants had schizophrenia. |
| (Warner, Huxley, & Berg, 1999) | 38 matched pairs | Observational Study comparing Clubhouse members to Matched Controls (matched on demographics and length of psychiatric service history) | Usual Clubhouse Services | 2 years | Quality of Life and Social Support | Clubhouse members had better mean scores for Quality of Life domains of finances, legal/safety and global well-being. Clubhouse members had a higher percentage reporting having close friends (92% to 62%) and having someone to rely upon when they needed help (100% to 63%). |
| (Weiss, Maddox, Vanderwaerden, & Szilvagyi, 2004) | 13 | Observational study | Tri-county Scholars program (enhanced educational training) | 1 year | Educational enrollment and employment | Mostly reported process outcomes. Out of 13 attendees, 1 had been enrolled before the program, 6 took classes after attending, 4 worked while attending the program, 10 worked following attending. |
| (Wilkinson, 1992) | 32 | Observational Study | Usual Clubhouse services | 8 years | Re-hospitalization | Comparing an equal amount of time for each participant (years prior to entering Clubhouse vs. years as a member), after becoming a member the average length of hospitalization decreased and the number of total hospitalizations decreased. |
| (Yau, Chan, Chan, & Chui, 2005) | 39 | Observational (New Clubhouse members compared to members who had already attended 3 months or more) | Usual Clubhouse services | 12 weeks (measured at baseline and again after 12 weeks of attendance) | Work related skills, coping skills, cognitive abilities, social functioning | Emotional coping and work personality were improved in new members after attending the Clubhouse program for 12 weeks (specific improvements in task orientation, social skills and team work). |

***(Met International Clubhouse Standards and Reported on Main Outcomes)**

References

1. Accordino, M. P., & Herbert, J. T. (2000). Treatment outcome of four rehabilitation interventions for persons with serious mental illness. *Journal of Mental Health Counseling, 22*(3), 268-282.
2. Adler, J. (1976). *A study of the relationship between the reality of self-perception of former mental patients at fountain house and their social and vocational adjustment. 37*, 945B.
3. Baker, S. (2013). *Determining appropriate outcome measures in a psychosocial rehabilitation model for the mentally ill: A knowledgeable citizens' perspective.* (Doctoral dissertation). Mississippi State University, Starkville, Mississippi.
4. Barry, P. C., II. (1982). *Selected neuropsychological and rehabilitation assessment measures with chronically mentally ill adults.*
5. Beard, J. H., Malamud, T. J., & Rossman, E. (1978). Psychiatric rehabilitation and long-term rehospitalization rates: The findings of two research studies. *Schizophrenia Bulletin, 4*(4), 622-635.
6. Beard, J. H., Pitt, R. B., Fisher, S. H., & Goertzel, V. (1963). Evaluating the effectiveness of a psychiatric rehabilitation program. *American Journal of Orthopsychiatry, 33*, 701-712.
7. Beckel, D. N. (1998). *Clozapine and clubhouse treatment model and vocational outcomes of adults with schizophrenia. Dissertation Abstracts International Section B: The Sciences & Engineering, 59(5-B)*, 1-93.
8. Biegel, D.E., Pernice-Duca, F., Chang, C.W., Chung, C.L., Min, M.O., & D'Angelo, L. (2013). *Family social networks and recovery from severe mental illness of clubhouse members.* Journal of Family Social Work, 16, 274-296.
9. Biegel, D.E., Pernice-Duca, F., Chang, C.W., & D'Angelo, L. (2013). Correlates of peer support in a clubhouse setting. Community Mental Health Journal, 48, 153-160.
10. Booth, M. E. (1994). *Effects of program factors on client outcomes: Evaluation of three clubhouse programs for persons with severe mental illness. DAI-A 54/12,* p. 4588.
11. Boyd, A. S., & Bentley, K. J. (2005). The relationship between the level of personal empowerment and quality of life among psychosocial clubhouse members and consumer-operated drop in center participants. *Social Work in Mental Health, 42*(2), 67-93.
12. Carolan, M., Onaga, E., Pernice-Duca, F., & Jimenez, T. (2011). A place to be: The role of clubhouses in facilitating social support. *Psychiatric Rehabilitation Journal, 35*(2), 125-132.
13. Crowther, R., Marshall, M., Bond, G.R., & Huxley, P. (2010). *Vocational rehabilitation for people with severe mental illness* (Rep. No. CD003080). John Wiley and Sons, Ltd.
14. Donnell, C. M. (2001). *The clubhouse model in michigan: A preliminary examination of individual and organizational characteristics associated with employment outcomes.* , 1-177.
15. Dorio, J., Guitar, A., Solheim, L., Dvorkin, C., & Marine, S. (2002). Differences in job retention in a supported employment program: Chinook clubhouse. *Psychiatric Rehabilitation Journal, 25*(3), 289-298..
16. Dougherty, S., Hastie, C., Bernard, J., Broadhurst, S., & Marcus, L. (1992). Supported education: A clubhouse experience. *Psychosocial Rehabilitation Journal, 16*(2), 91-104.
17. Gold, P.B., Macias, C., & Rodican, C.F. (2016). Does competitive work improve quality of life for adults with severe mental illness? Evidence from a randomized trial of supported employment. *The Journal of Behavioral Health Services & Research*, 43, 155-171.
18. Gregitis, S., Glacken, J., Julian, C., & Underwood, K. (2010). Comparing working role values of employed and unemployed clubhouse members. *Work, 36*, 39-46.
19. Grinspan, Z. (2015). *Brief research report: Fountain House and use of healthcare resources.* Weill Cornell Medical College, New York, NY.
20. Gumber, S. (2011). *Living in the community with serious mental illness: Community integration experiences of clubhouse members.* Retrieved from ProQuest Dissertations and Theses. Accession Order No. 3493140.
21. Hancock, N., Honey, A., & Bundy, A.C. (2015). *Sources of meaning derived from occupational engagement for people recovering from mental illness.* British Journal of Occupational Therapy, 78, 508-515.
22. Henry, A. D., Barreira, P., Banks, S., Brown, J., & McKay, C. (2001). A retrospective study of clubhouse-based transitional employment. *Psychiatric Rehabilitation Journal, 24*(4), 344.
23. Henry, A., Jackson, R., Fisher, W., Brown, J. M., & White, A. (1999). *A retrospective examination of the relationship of clubhouse membership to clinical service use.* A report to the International Center for Clubhouse Development. University of Massachusetts Medical School, Worcester, MA. (Unpublished Manuscript).
24. Jacobs, D. R. (1999). *An effectiveness study of psychosocial rehabilitation.* The Union Institute Graduate School, US). *Dissertation Abstracts International Section B: The Sciences & Engineering, 60*(10), 1-86.
25. Jacobs, H. E., & DeMello, C. (1996). The clubhouse model and employment following brain injury. *Journal of Vocational Rehabilitation, 7*(3), 169-179.
26. Johnsen, M., McKay, C., Henry, A., & Manning, T. D. (2004). *What does competitive employment mean? A secondary analysis of employment approaches in the Massachusetts Employment Intervention Demonstration Project*. In W. Fisher (Ed.), Research in Community and Mental Health, Volume 13. Research on Employment for Persons with Severe Mental Illness. (pp. 43‐62). Amsterdam: Elsevier, JAI.
27. Jung, S.H. & Kim, H.J. (2012). Perceived stigma and quality of life of individuals diagnosed with schizophrenia and receiving psychiatric rehabilitation services: A comparison between the clubhouse model and a rehabilitation skills training model in South Korea. *Psychiatric Rehabilitation Journal*, 35, 460-465.
28. Kelliher, S. (2006). *Factors influencing member employment in international center for clubhouse development (ICCD) clubhouses. 67(2-B)*, 1152. (yes). (Dissertation Abstract: 2006-99016-021).
29. Macias, C., Barriera, P., Alden, M., & Boyd, A. S. (2001). The ICCD benchmarks for clubhouses: A practical approach to quality improvement in psychiatric rehabilitation. *Psychiatric Services, 52*(2), 207-213.
30. Macias, C., DeCarlo, L. T., Wang, Q., Frey, J., & Barreira, P. (2001). Work interest as a predictor of competitive employment: Policy implications for psychiatric rehabilitation. *Administration and Policy in Mental Health, 28*(4), 279-297.
31. Macias, C., Kinney, R., & Rodican, C. (1995). Transitional employment: An evaluative description of fountain house practice. *Journal of Vocational Rehabilitation, 5*, 151-157.
32. Macias, C., Rodican, C. F., Hargreaves, W. A., Jones, D. R., Barreira, P. J., & Wang, Q. (2006). Supported employment outcomes of a randomized controlled trial of ACT and clubhouse models. *Psychiatric Services, 57*(10), 1406-1415.
33. Malamud, T. J., & McCrory, D. (1988). Transitional employment and psychosocial rehabilitation. In J. A. Cardiello, & M. D. Bell (Eds.), (pp. 150-162). Baltimore, MD: Johns Hopkins University Press.
34. McKay, C., Johnsen, M., & Stein, R. (2005). Employment outcomes in massachusetts clubhouses. *Psychiatric Rehabilitation Journal, 29*(1), 25-33.
35. McKay, C. E., Johnsen, M., Banks, S., & Stein, R. (2006). Employment transitions for clubhouse members. *Work.26(1):67-74, 26*(1), 67-74.
36. Mowbray, C. T., Holter, M. C., Mowbray, O. P., & Bybee, D. (2005). Consumer-run drop-in centers and clubhouses: Comparisons of services and resources in a statewide sample. *Psychological Services, 2*(1), 54-64.
37. Mowbray, C. T., Woodward, A. T., Holter, M. C., MacFarlane, P., & Bybee, D. (2009). Characteristics of users of consumer-run drop-in centers versus clubhouses. *Journal of Behavioral Health Services & Research, 36*(3), 361-371.
38. Okon, S., Webb, D., Zehnder, E., Kobylski, M., Morrow, C., Reid, V. et al. (2015). Health and wellness outcomes for members in a psychosocial rehabilitation clubhouse participating in a healthy lifestyle design program. Occupational Therapy in Mental Health, 31, 62-81.
39. Pelletier, J. R., Nguyen, M., Bradley, K., Johnsen, M., & McKay, C. (2005). A study of a structured exercise program with members of an ICCD certified clubhouse: Program design, benefits, and implications for feasibility. *Psychiatric Rehabilitation Journal, 29*(2), 89-96.
40. Pernice-Duca, F. M. (2008). The structure and quality of social network support among mental health consumers of clubhouse programs. *Journal of Community Psychology, 36*(7), 929-946.
41. Reed, S. J., & Merz, M. A. (2000). Integrated service teams in psychiatric rehabilitation: A strategy for improving employment outcomes and increasing funding. *Journal of Applied Rehabilitation Counseling, 31*(4), 40-46.
42. Rosenfield, S. & Neese-Todd, S. (1993). Elements of a psychosocial clubhouse program associated with a satisfying quality of life. *Hospital & Community Psychiatry*, 44, 76-78.
43. Schonebaum, A. D., Boyd, J. K., & Dudek, K. J. (2006). A comparison of competitive employment outcomes for the clubhouse and PACT models. *Psychiatric Services, 57*(10), 1416-1420.
44. Schonebaum, A., & Boyd, J. (2012). Work-ordered day as a catalyst of competitive employment success. article]. *Psychiatric Rehabilitation Journal, 35*(5), 391-395.
45. Spence, B. (2014). *Social network and social support at a clubhouse program.* The Ohio State University, Ohio. Retrieved from: <http://hdl.handle.net/1811/60416>
46. Stein, L., Lawton Barry, K., Van Dien, G., Hollingsworth, E. J., & Sweeney, J. K. (1999). Work and social support: A comparison of consumers who have achieved stability in ACT and clubhouse programs. *Community Mental Health Journal, 35*(2), 193-204.
47. Tsang, A.W.K., Ng, R.M.K., & Yip, K.C. (2010). A six-month prospective case-controlled study of the effects of the clubhouse rehabilitation model on Chinese patients with chronic schizophrenia. *East Asian Archives of Psychiatry*, 20, 23-30.
48. Unger, K.V. & Pardee, R. (2002). Outcome measures across program sites for postsecondary supported education programs. *Psychiatric Rehabilitation Journa*l, 25, 299-303.
49. Warner, R., Huxley, P., & Berg, T. (1999). An evaluation of the impact of clubhouse membership on quality of life and treatment utilization. *International Journal of Social Psychiatry, 45*(4), 310-320.
50. Weiss, J., Maddox, D., Vanderwaerden, M., & Szilvagyi, S. (2004). The tri-county scholars program: Bridging the clubhouse and community college. *American Journal of Psychiatric Rehabilitation, 7*(3), 281-300.
51. Wilkinson, W. (1992). New Day, Inc. of Spartanburg: Hospitalization study. *Psychosocial Rehabilitation Journal, 16*(2), 163-168.
52. Yau, E. F., Chan, C. C., Chan, A. S., & Chui, B. K. (2005). Changes in psychosocial and work-related characteristics among clubhouse members: A preliminary report. *Work, 25*(4), 287-296.
